# New Tools for Epilepsy Therapy

**DOI:** 10.3389/fncel.2018.00147

**Published:** 2018-05-29

**Authors:** Chiara Falcicchia, Michele Simonato, Gianluca Verlengia

**Affiliations:** ^1^Department of Medical Sciences, Section of Pharmacology, and Neuroscience Center, University of Ferrara and National Institute of Neuroscience, Ferrara, Italy; ^2^School of Medicine, University Vita-Salute San Raffaele, Milan, Italy

**Keywords:** epilepsy, cell therapy, delivery devices, gene therapy, herpes-based vector

## Abstract

One third of the epilepsies are refractory to conventional antiepileptic drugs (AEDs) and, therefore, identification of new therapies is highly needed. Here, we briefly describe two approaches, direct cell grafting and gene therapy, that may represent alternatives to conventional drugs for the treatment of focal epilepsies. In addition, we discuss more in detail some new tools, cell based-biodelivery systems (encapsulated cell biodelivery (ECB) devices) and new generation gene therapy vectors, which may help in the progress toward clinical translation. The field is advancing rapidly, and there is optimism that cell and/or gene therapy strategies will soon be ready for testing in drug-resistant epileptic patients.

## Epilepsy: Therapeutic Needs

Epilepsies are a heterogeneous group of disorders whose single common symptom is the unpredictable occurrence of seizures. In addition to seizures, people with epilepsy have an increased risk of early death, can experience cognitive, neurological and psychiatric comorbidities, and overall suffer a significantly lower quality of life than the rest of the population. Altogether, the epilepsies are very common neurological disorders, affecting tens of millions of people worldwide. Unfortunately, conventional antiepileptic drugs (AEDs) control seizures in only about two thirds of the cases, and do not exert any effect on epilepsy co-morbidities. Therefore, a major and urgent challenge is to develop new antiepileptic treatments.

The new drugs released into clinical practice over the past few years have not substantially changed this situation (Simonato et al., 2014), and non-pharmacological alternatives (chetogenic diet, vagal nerve stimulation, deep brain stimulation) proved to have limited efficacy. One effective therapeutic option for the management of drug refractory epilepsy is resective brain surgery. However, this procedure can only be considered for focal epilepsies, is complex and costly, and is often hampered by obstacles (like vicinity to eloquent brain areas) which prevent its application to many patients who may benefit from it.

Advanced therapies (like gene therapy, cell therapy or even a combination of both) may become another option for drug-resistant focal epilepsies, that may be offered as an alternative to surgery or even when surgery is precluded. In this short review, we will discuss these new approaches, with emphasis on their limitations and strategies to overcome these limitations and facilitate clinical translation.

## Cell Therapy Approaches: Direct Cell Graft Injection

A promising treatment option, particularly for focal epilepsies, could be the direct grafting of cells. Björklund and Lindvall (2000) were the first to evaluate the effects of neural transplantation in epilepsy models. These strategies have received considerable attention because the transplanted cells can survive and sometimes can even become structurally and functionally integrated into the epileptic brain. Cells may be transplanted to replace lost cells or to release substances that modulate existent hyper-excitability (so-called by-stander effect). A successful cell graft should allow cells to survive, generate the desired phenotypes, migrate to the correct position, establish appropriate and functional integration into neural networks. Alternatively, it should survive, produce the desired therapeutic factor and release it long-term. In addition, grafts should not produce any adverse effect, while some studies report unwanted side effects, including paradoxical generation of seizures (Löscher et al., 2008).

As shown in Table 1, a variety of cell types have been tested in animal models of epilepsy, including neural stem cells; human induced pluripotent stem cells; GABAergic progenitors derived from mouse or human embryonic stem cells; GABAergic precursors; striatal precursor cells (Hattiangady et al., 2008; Baraban et al., 2009; Gallego et al., 2010; De la Cruz et al., 2011; Shetty and Upadhya, 2016). Several studies now convincingly support the notion that some types of cells can successfully treat epilepsy in animal models. For example, Thompson (2005) demonstrated the seizure-suppressing capabilities of engineered cells with regulatable GABA production transplanted into the dentate gyrus in the kindling model (Thompson, 2005). In another study, adenosine releasing embryonic mouse stem cells, when differentiated into neural precursor cells and transplanted to the hippocampus, produced a long-term suppression of kindled seizures (Li et al., 2007). While important from a theoretical point of view, the limit of these studies is that treatment was applied before seizure-eliciting stimulations, i.e., under conditions not immediately translatable to the human situation. In the successive studies described below treatment was more appropriately applied when animals were already experiencing spontaneous recurrent seizures.

**Table 1:** Cell therapy in animal models of epilepsy.

Model and species	Type of treatment	Period of treatment	Outcome	Reference
Genetically epilepsy-prone rats	Fetal raphe tissue in the third ventricle	Chronic pre-disposition to seizures	Reduced audiogenic-induced seizures severity	Clough et al. (1996)
Kindling (rat)	Cholinergic neurons in the hippocampus	Before kindling	Delayed kindling development	Ferencz et al. (1998)
Kindling (rat)	Embryonic striatal GABAergic neurons in the substantia nigra	Fully kindled	Transiently reduced seizure severity	Löscher et al. (1998)
Kindling (rat)	Immortalized neurons engineered to produce GABA in the dentate gyrus	Before kindling	Increased after-discharge threshold and reduced after-discharge duration	Thompson (2005)
Kainic acid (rat)	Hippocampal fetal cells pre-treated with neurotrophic factors in the hippocampus	Chronic period	Reduced seizure frequency	Rao et al. (2007)
Kindling (rat)	Adenosine-releasing neural precursor cells in the hippocampus	Before kindling	Delayed kindling development	Li et al. (2007)
Kainic acid (rat)	Striatal precursor cells in the hippocampus	Latency	Reduced seizure frequency	Hattiangady et al. (2008)
Kv1.1 mutant mouse	Precursor cells from the medial ganglionic eminence in cortex	Before the beginning of spontaneous seizures	Reduced seizure duration and frequency	Baraban et al. (2009)
Kindling (rat)	Embryonic median ganglionic eminence cells in the basolateral amygdala	After kindling	Increased after-discharge threshold	Gallego et al. (2010)
Kindling (rat)	Fibroblasts in the basolateral amygdala	After kindling	Increased after-discharge threshold	Gallego et al. (2010)
4-aminopyridine (mouse)	Interneuron progenitors from medial ganglionic eminence in the motor cortex	Before 4-amino-pyridine	Attenuated power of focal ictal discharges	De la Cruz et al. (2011)
Pilocarpine (mouse)	Medial ganglionic eminence GABA progenitors in the hippocampus	Chronic period	Reduced seizure frequency and improved behavior	Hunt et al. (2013)
Kindling (rat)	ARPE-19 galanin secreting cells in the hippocampus	Before kindling	Moderate suppression of stimulation-induced seizures	Nikitidou et al. (2014)
Systemic pilocarpine (mouse)	Cells from medial ganglionic eminence in the dentate gyrus	Chronic period	Transient reduced seizure frequency and severity	Henderson et al. (2014)
Systemic pilocarpine (mouse)	GABAergic interneurons derived from human pluripotent stem cells in the hippocampus	Chronic period	Seizure suppression and improvement in behavioral co-morbidities	Cunningham et al. (2014)
Pilocarpine (mouse)	Progenitor cells from embryonic medial or caudal ganglionic eminence in the hippocampus	Chronic period	Reduced seizure frequency	Casalia et al. (2017)
Pilocarpine (rat)	ARPE-19 BDNF secreting cells in the hippocampus	Chronic period	Reduced seizure frequency, improved cognitive performance, and reversal of histological alterations	Falcicchia et al. (2018)

Maybe the most compelling evidence of efficacy of a cell transplant in epilepsy models was provided by Baraban et al. (2009) using medial ganglionic eminence (MGE) GABA progenitors. In their seminal paper (Hunt et al., 2013) they found that transplantation of these cells into the hippocampus (but not into the amygdala) of adult epileptic mice markedly reduced occurrence of seizures as well as deficits in learning and aggressive behavior. They subsequently demonstrated that these effects can last long term (more than 6 months; Casalia et al., 2017). In addition, MGE (but not caudal ganglionic eminence, CGE) progenitors, when transplanted into a neonatal hippocampus, can migrate, differentiate into mature interneurons, and form appropriate inhibitory synaptic connections with pyramidal neurons (Hsieh and Baraban, 2017).

Altogether, these studies demonstrate that grafting specific cell types in the brain can result not only in a reduction of seizure frequency, but also in the improvement of co-morbidities of epilepsy. However, a number of issues remain to be addressed before attempting clinical translation of cell grafting to human epilepsy. For instance, a more in-depth knowledge of the fate of grafted cells post-transplantation is needed: How far can they migrate? How long do they survive? How to they affect the function of neuronal networks? As described above, these questions have been challenged in rodent models, but more knowledge and scale-up in larger brains are needed before proceeding to the clinics. Moreover, there are still issues associated with the possibility that grafted cells may induce immunological reactions. Finally, a key problem is identifying a rescue strategy in case of adverse effects, because these cell grafts cannot be removed once injected (Emerich et al., 2014).

## Novel Cell-Based Biodelivery Systems

**Figure 1:**
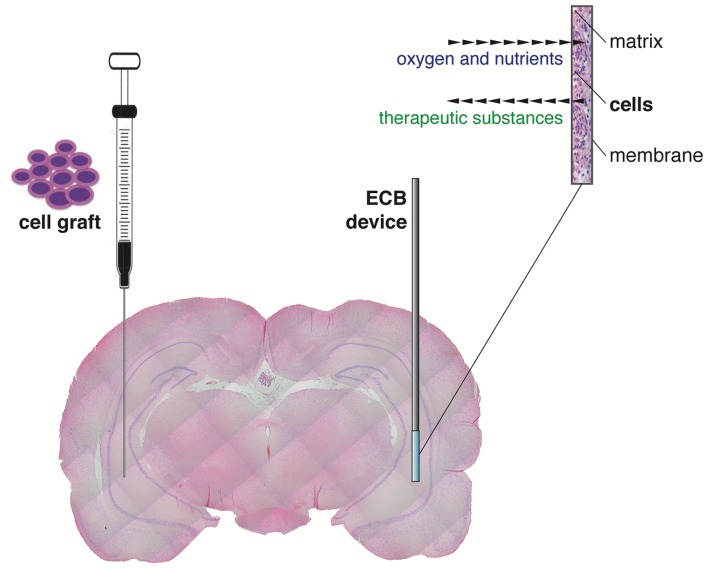
Graphical representation of the “classic” direct cell graft injection (*left*) and the novel cell-based biodelivery system (encapsulated cell biodelivery (ECB), *right*). The semipermeable membrane of the ECB devices allows the influx of oxygen and nutrients and the outflow of the therapeutic substances, with no need of immunosuppression.

An alternative and reliable approach to delivery therapeutic molecules (i.e., to pursue a by-stander effect, not an integration of cells in the circuitry), directly to the epileptic brain region of interest across blood brain barrier, is the encapsulated cell biodelivery (ECB) system. This technology targets diseased neurons with therapeutic biological substances that are continuously produced and secreted by genetically engineered human cells enclosed within a device (Emerich et al., 2014). More in detail, cells are engineered to produce the desired therapeutic substance and then encapsulated in a biocompatible matrix coated with a thin polymer membrane. Therefore, when inserted in the target brain area, cells are kept separated from the adjacent host brain tissue (Figure 1). The membrane admits oxygen and required nutrients from the tissue to nourish cells, while restricting passage of larger cytotoxic agents from the host immune defense system, avoiding the need of immunosuppression. By using human cells as the delivery vehicle, the chances of immunological reactions are even further reduced. At the same time, the therapeutic substance released by the engineered cells can diffuse from the device into the surrounding tissue (Figure 1). This technology can be used to deliver essentially any cell-derived therapeutic, including recombinant growth factors, peptides and antibodies. Importantly, ECB devices can be easily and safely retrieved in case of unwanted effects.

ECB devices secreting neuropeptides (galanin) or neurotrophic factors (glial cell-derived neurotrophic factor, GDNF and brain-derived neurotrophic factor, BDNF) have been tested in chronic animal models of epilepsy, leading to reduction in the frequency of seizures, improvements in cognitive performance, and reversal of histological alterations associated with chronic epilepsy (Kanter-Schlifke et al., 2009; Nikitidou et al., 2014; Falcicchia et al., 2018). Because the ECB device approach has been already scaled-up in large animal brains and tested in humans with Alzheimer disease (Wahlberg et al., 2012), there is optimism on the possibility of starting soon clinical studies in epileptic patients with focal, drug-resistant epilepsy.

## Gene Therapy Approaches

After the first approved protocol for a human “gene transplant” in 1989 (Rosenberg et al., 1990), gene therapy went through changing fortunes that reverted the initial enthusiasm into scepticism. However, it eventually made great progress in the past few years that allowed some clinical applications and made other applications a more realistic perspective, as demonstrated by the increasing number of clinical trials with evidence of effectiveness (Carvalho et al., 2017). The most challenging goal for gene therapy remains the treatment of disorders affecting the central nervous system (CNS), even if promising results are coming from preclinical and initial clinical trials (Simonato et al., 2013; Kantor et al., 2014; Mendell et al., 2017). Advances have been made also in the field of epilepsy, particularly for application in focal epilepsies (reviewed by Kullmann et al., 2014; Simonato, 2014). Indeed, gene therapy (i.e., the direct *in vivo* administration of an engineered DNA-delivery vector into the diseased brain area) seems less invasive and troublesome compared to cell therapy procedures.

The first (but not the only) key issue to address in order to implement gene therapy in the clinics is to develop a vector system with the essential features for delivery into the CNS: (1) absence of risk due to insertional mutagenesis or viral DNA rearrangement; (2) no induction of cytoxicity or immune response; (3) high transduction efficiency; (4) robust and stable expression of the transgene, with a long-term effect after administration of a single dose; (5) regulated transgene expression; (6) cell type specificity, to infect exclusively the target cells and minimize off-target effects; (7) large packaging capacity, i.e., efficient methods to deliver multiple, independently regulated genes to the target cells; and (8) well characterized, reproducible and scalable methods of manufacturing, allowing to obtain high functional titres of pure vector stocks without contamination from helper viruses. In addition, the ability to cross the blood-brain-barrier would be needed for diseases affecting the whole brain.

In the past few years, the use of non-viral vectors has made a big step up for both *in vitro* and *in vivo* applications (Hardee et al., 2017). If compared to their viral counterpart, these tools are deemed to be generally safer, cheaper and relatively easier to produce. However, their employment for *in vivo* CNS targeting is still hindered by inadequate efficiency of transduction and transient expression of the transgene. At least so far, there is no evidence of efficacy for epilepsy treatment in preclinical or clinical studies by delivery of nucleic acids through non-viral systems. There is indeed a general consensus in recognizing virus-based vectors as the most convenient tools for an efficient delivery of the transgenes *in vivo* and for a stable and sustained expression in the target cells (Chira et al., 2015). Deceptively simple in its structure, a virus can be engineered by deletion of key elements from its genome, turning it harmless but still able to efficiently deliver nucleic acids in the target cells while making room for therapeutic genes. A wide variety of viruses have been adapted for gene transfer in the CNS (including lentiviruses, adenoviruses, adeno-associated viruses (AAVs) and herpes viruses), each with advantages and limitations.

Adenoviral vectors can be easily produced at high titres, an essential requirement for an *in vivo* administration, even with the insertion of a discrete-sized exogenous DNA (up to 8 kb). These vectors are able to infect dividing and non-dividing cells, their DNA is normally maintained as an episome and transgenes are promptly expressed (peak at 24–48 h). However, the residual cytotoxicity and other severe drawbacks, like the transient expression of the transgene and the acute inflammatory response (Liu and Muruve, 2003), have seriously questioned the appropriateness of adenoviral-based vectors for CNS applications. These drawbacks have been partially overcome by the latest generation of gutless adenoviral vectors, which have been deleted of much of the viral coding sequence. As a result, the packaging capacity of this adenoviral backbone raised up to 35 kb and, although it still causes some acute inflammation upon CNS injection, the toxicity has been significantly lowered and the expression of delivered transgenes is detectable up to 1 year (Soudais et al., 2004).

The most commonly used class of viruses for CNS targeting are the AAVs. AAVs can efficiently infect both dividing and non-dividing cells and ensure a prolonged expression of the transgene (Fischer et al., 2016; Kunze et al., 2018). The broad range of potentially infected cells is due to availability of a large array of capsid variants, each characterized by a diverse antigen profile (Gao et al., 2005). Some of these variants can even allow the vector to cross the blood-brain barrier, disclosing the possibility of an intravenous administration for CNS targeting (Deverman et al., 2016), an approach that was recently successfully used to treat children with spinal muscular atrophy (Mendell et al., 2017). The AAV platform offers the advantage of a relative safe profile, with negligible inflammatory responses and minimal integration into the host genome (Bessis et al., 2004). However, AAV has a very low packaging capacity for exogenous DNA (<5 kb), unsuitable for many gene therapy approaches that require large-sized expression cassettes (Loring et al., 2016).

Unlike AAVs, lentiviruses integrate their DNA in the host cell genome. This feature ensures a stable and prolonged transgene expression, not affected to dilution with cell division (Benskey and Manfredsson, 2016). However, the downside is the possibility of getting insertional mutagenesis (Bokhoven et al., 2009). To overcome this problem, a new class of lentiviruses has been generated to confer a steady expression of the transgene after *in vivo* infection in neurons *in vivo* without integration (Yáñez-Muñoz et al., 2006). Relatively easier to produce compared to AAVs, the larger size of lentiviruses allows for bigger DNA insertions (up to 9 kb).

## New Gene Therapy Vectors

Replication-defective herpes simplex virus type 1 (HSV-1) based vectors are attractive tools for neurological gene therapy because of the unique feature of offering a large payload capacity, theoretically up to 30–40 kb. The herpetic DNA does not integrate into the host DNA, the viral genes being expressed from an episomal form of the genome. This feature implies the absence of risk of insertional mutagenesis. Moreover, HSV-1 has a remarkable ability to infect neurons and to establish a lifelong non-toxic latency, during which neurospecific regulatory elements confer a sustained expression of specific genes termed latency-associated transcripts (LATs). In the lytic phase, the transcription of viral genes is temporally regulated in a cascade manner. The first to be expressed upon infection are the immediate early (IE) genes; these genes undergo transcription in the absence of *de novo* protein synthesis. Through single or multiple IE genes deletion and the removal of additional non-essential regions, HSV-1 based vector can be made unable to replicate unless in complementing cell lines (Grant et al., 2009). Attempts of gene therapy treatments for CNS disorders have been carried out with replication-defective HSV vectors (Paradiso et al., 2009) but, so far, residual neuronal cytotoxicity and loss of transgene expression within the first few weeks after infection have seriously hindered their development. A critical problem is that, while the toxicity is mainly caused by the expression of ICP0, an IE protein that alters chromatin structure and mediates an host immune response escape, the deletion of ICP0 rapidly shuts down transgene expression (Cliffe and Knipe, 2008).

**Figure 2:**
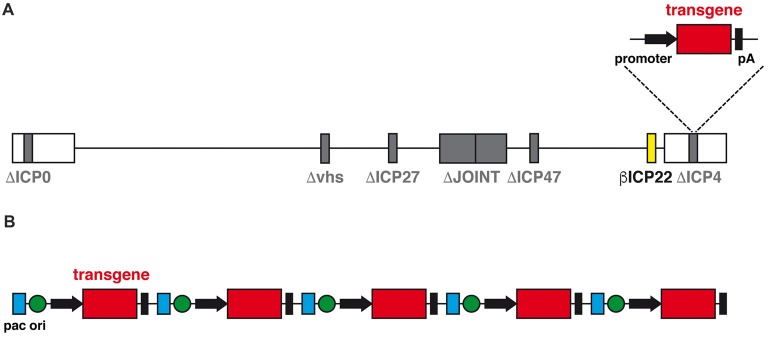
**(A)** Schematic diagram of the linear genomic structure of JΔNI8, the latest generation of replication-defective HSV-1 based vector. The whole JOINT region is deleted, along with the genes coding for ICP0, ICP4, ICP27 and vhs (in gray). The expression of ICP22 is converted from immediate early (IE) to early-like kinetic by promoter modification (in yellow). A transgene expression cassette is subcloned into the ICP4 locus. **(B)** Schematic representation of the amplicon vector genome, consisting in a concatamer of multiple copies of the amplicon plasmid, each with an origin of replication (ori, green), an HSV-1 packaging (pac, blue) and a transgene expression cassette.

The advent of a new generation of HSV-1 based vector may change the perspectives of use of herpes vectors as a safe and worthwhile gene delivery tool. This new family of vectors is functionally deleted of all IE genes, which prevents expression of any viral protein and, therefore, abolishes any residual cytotoxicity; however, transgene expression in both neuronal and non-neuronal cells *in vitro* is not compromised (Miyagawa et al., 2015). In addition, at variance with the previous results mentioned above, the deletion of ICP0 does not prevent transgene expression from a specific locus, ICP4. When the expression cassette was inserted in ICP4, a robust, sustained and neuron-specific transgene expression was observed in several rat brain regions, and this was clearly detectable for at least 6 months after vector injection with no detectable sign of neuronal toxicity or immune response (Verlengia et al., 2017). A further refinement of this vector was obtained through the additional removal of the virion host shutoff (vhs) gene (Figure 2A), a strategy that further increased both the levels of transgene expression in neurons and the safety profile of the vector (Miyagawa et al., 2017).

Another alternative vector system is represented by the amplicons (Figure 2B). Amplicon vectors are identical to the wild type HSV-1 in their structural, immunological and host range features but they differ in the genome, that contains an origin for DNA replication in bacteria and the bare minimum HSV viral packing signals for vector propagation in cells co-infected with a defective HSV helper virus (Simonato et al., 2000). These minimal sequences allow for incorporation of up to 150 kb of foreign DNA; no other vector can come even close to this huge packaging capacity (Kwong and Frenkel, 1984). The absence of almost any viral gene also protects from the risk of recombination with potential latent HSV-1 genomes *in vivo* and strongly limits the cytotoxic effects. The downside of the system is the need to use helper viruses for the preparation of the vector; although these can be effectively removed, even a contamination as low as 1% may be risky and unacceptable for testing in humans. While waiting for a solution to this problem, amplicon vectors already represent a wonderful tool for testing the effects of locally-delivered therapeutic proteins (Falcicchia et al., 2016).

In sum, these new delivery tools, highly replication-defective HSV-based vectors and amplicon vectors, may become a realistic option for gene therapy treatments of CNS disorders in which a restricted brain area must be targeted, like in focal epilepsies.

## Author Contributions

CF, MS and GV contributed to the literature search and to the writing of this review.

## Conflict of Interest Statement

The authors declare that the research was conducted in the absence of any commercial or financial relationships that could be construed as a potential conflict of interest.
