# Corrigendum: New Tools for Epilepsy Therapy

**DOI:** 10.3389/fncel.2018.00212

**Published:** 2018-07-11

**Authors:** Chiara Falcicchia, Michele Simonato, Gianluca Verlengia

**Affiliations:** ^1^Department of Medical Sciences, Section of Pharmacology, and Neuroscience Center, University of Ferrara and National Institute of Neuroscience, Ferrara, Italy; ^2^School of Medicine, University Vita-Salute San Raffaele, Milan, Italy

**Keywords:** epilepsy, cell therapy, delivery devices, gene therapy, herpes-based vector

In the original article, we made the incorrect statement that non-viral vectors do not work *in vivo*, as below:

“Although the use of non-viral vectors could be attractive as they are generally safe, cheap and relative easy to produce, these approaches do not work *in vivo*”

This mistake was due to the fact that we meant to refer to epilepsy. Non-viral vectors have provided promising results for other applications, even if less for central nervous system disorders and in no case for epilepsy.

A correction has been made to section “Gene Therapy Approaches”, Paragraph 3:

In the past few years, the use of non-viral vectors has made a big step up for both *in vitro* and *in vivo* applications (Hardee et al., 2017). If compared to their viral counterpart, these tools are deemed to be generally safer, cheaper and relatively easier to produce. However, their employment for *in vivo* CNS targeting is still hindered by inadequate efficiency of transduction and transient expression of the transgene. At least so far, there is no evidence of efficacy for epilepsy treatment in preclinical or clinical studies by delivery of nucleic acids through non-viral systems.

The authors apologize for this error and state that this does not change the scientific conclusions of the article in any way.

The original article has been updated.

## Conflict of interest statement

The authors declare that the research was conducted in the absence of any commercial or financial relationships that could be construed as a potential conflict of interest.
